# Insights from a national survey in 2021 and from modelling on progress towards hepatitis C virus elimination in the country of Georgia since 2015

**DOI:** 10.2807/1560-7917.ES.2023.28.30.2200952

**Published:** 2023-07-27

**Authors:** Josephine G. Walker, Irina Tskhomelidze, Shaun Shadaker, Maia Tsereteli, Senad Handanagic, Paige A. Armstrong, Amiran Gamkrelidze, Peter Vickerman

**Affiliations:** 1 Population Health Sciences, University of Bristol, Bristol, United Kingdom; 2 Task Force for Global Health, Tbilisi, Georgia; 3 Division of Viral Hepatitis, Centers for Disease Control and Prevention, Atlanta, United States; 4 National Center for Disease Control and Public Health of Georgia, Tbilisi, Georgia

**Keywords:** viral hepatitis, elimination, modelling, program evaluation

## Abstract

**Background:**

Between May 2015 and February 2022, 77,168 hepatitis C virus (HCV)-infected people in Georgia have been treated through an HCV elimination programme. To project the programme’s long-term impacts, an HCV infection model was initially developed, based on data from surveys among people who inject drugs and a national serosurvey in 2015.

**Aim:**

Accounting for follow-up surveys in 2021, we validate and update projections of HCV infection prevalence and incidence.

**Method:**

We assessed the initial model projections’ accuracy for overall prevalence, by age, sex, and among people who ever injected drugs, compared with 2021 serosurvey data. We used 2021 results to weight model fits and to recalculate the national programme’s impact leading up to March 2022 on HCV infection incidence rates. Cases and deaths averted were estimated. The impact of reduced treatment rates during the COVID-19 pandemic was assessed.

**Results:**

The original model overpredicted adult (≥ 18 years old) chronic HCV infection prevalence for 2021 (2.7%; 95% credible interval (CrI): 1.9–3.5%) compared with a 2021 serosurvey (1.8%; 95% confidence interval (CI): 1.3–2.4%). Weighted model projections estimated a 60% decrease in HCV infection incidence by March 2022, with an absolute incidence of 66 (95% CrI: 34–131) per 100,000 person-years (overall population). Between May 2015 and March 2022, 9,186 (95% CrI: 5,396–16,720) infections and 842 (95% CrI: 489–1,324) deaths were averted. The COVID-19 pandemic resulted in 13,344 (95% CrI: 13,236–13,437) fewer treatments and 438 (95% CrI: 223-744) fewer averted infections by March 2022.

**Conclusion:**

Results support the programme’s high effectiveness. At current treatment rate (406/month), 90% reductions in prevalence and incidence in Georgia are achievable by 2030.

**Key public health message**

**What did you want to address in this study?**

Serosurveys evaluated hepatitis C virus (HCV) infection prevalence in 2015 and 2021 in the country of Georgia. We updated a dynamic model of HCV transmission in the general population and people who inject drugs (PWID), to match prevalence in both serosurveys. Using this updated model, we sought to evaluate the progress towards HCV elimination in terms of reductions in HCV infection incidence.

**What have we learnt from this study?**

Based on the updated model of HCV infection, we found a decrease of incidence of approximately 60% between 2015 and 2022 in both the general population and PWID. If treatment rates are maintained or increased, 90% reductions in prevalence and incidence can occur by 2030. Absolute incidence reduction elimination targets set by the World Health Organization (WHO) for 2030 are already met in PWID but not in the general population.

**What are the implications of your findings for public health?**

Georgia’s HCV elimination programme has been highly effective, reducing prevalence and incidence by more than half in 6 years, and WHO elimination targets are likely to be met by 2030 if treatment rates continue.

## Introduction

The country of Georgia had a high prevalence of hepatitis C virus (HCV) infection in 2015, with 5.4% of adults (aged ≥ 18 years) having a viraemic infection (150,340 people (95% confidence interval (CI): 128,060 to 173,060) of a population of ca 3.7 million) [1], compared with a global average of 1.0% in the overall population [2]. Due to the high risk of bloodborne virus transmission, the prevalence and incidence of HCV infection are often high in populations of people who inject drugs (PWID). The prevalence of viraemia due to HCV in PWID was estimated to be 39.2% in 2015 globally, and 51.2% in Georgia [3]. In 2016, the World Health Organization (WHO) launched the Global Health Sector Strategy for Viral Hepatitis which included targets for the elimination of HCV infection as a public health threat, defined as an 80% reduction in incidence of HCV infection and 65% reduction in mortality by 2030, or absolute targets of annual incidence < 5 per 100,000 person-years in the general (not PWID) population and two per 100 person-years in PWID [4]. Prior to this, Georgia had launched a national elimination programme in 2015 with a target of reducing chronic hepatitis C prevalence by 90%, to be achieved by scaling up testing and treatment alongside prevention measures in both the general population and PWID [5].

The baseline for the HCV elimination programme in Georgia was a national serosurvey conducted in 2015, which found a large variation in anti-HCV prevalence by age group and sex [1]. Men aged 40–49 years had the highest prevalence, with 22.7% anti-HCV positive, while in women anti-HCV prevalence increased with age to a high of 5.4% in those 60 years or older [1]. The serosurvey also identified reported history of injecting drug use (IDU) and blood transfusion as key risk factors for exposure to HCV, with adjusted odds ratios (OR) of 21.4 (95% CI: 12.3 to 37.4) and 4.5 (95% CI: 2.8 to 7.2), respectively. To evaluate progress towards achieving elimination, a follow up serosurvey was conducted in 2021, which found a 67% reduction in chronic hepatitis C prevalence, for a viraemic prevalence of 1.8% (95% CI: 1.3 to 2.4%) among adults (aged ≥ 18 years) [6]. This massive progress was achieved partly through a considerable scale up of screening and treatment access, resulting in more than 76,000 persons initiating treatment between 2015 and 2021 [7].

After the launch of the HCV elimination programme, we developed a dynamic mathematical model for HCV transmission in Georgia to project when the elimination targets for reductions in prevalence, incidence, and mortality would be met [8]. Our model, which accounted for actual treatment numbers through February 2019, projected that if treatment levels continued at a rate of 1,000 patients per month, as was the average during 2015–2018 the prevalence and incidence of HCV infection would be reduced to half by the end of 2020 [8]. This corresponded to a projected decrease in prevalence of 56%, with 95% credible interval (CrI; i.e. the intervals between 2.5% and 97.5% quantiles) of 46 to 67%, by the time of the serosurvey in 2021. Despite overall lower treatment initiation rates during the COVID-19 pandemic in 2020–2021 [9], a larger reduction in prevalence was observed.

In this analysis, we use the results of the 2021 serosurvey to re-evaluate and validate our model projections and identify how the model could be modified to better fit the new data. We then produce new estimates of the ongoing impact of the elimination initiative on hepatitis C incidence, infections averted, and deaths.

## Methods

### Serosurvey

The 2021 serosurvey used a stratified, multi-stage cluster design with systematic sampling, with a sample size target of 8,010 adults (aged ≥ 18 years), and 2,692 children aged 5–17 years. The country was divided into 10 strata and 267 clusters, with 30 households per cluster chosen systematically. In each household, one adult and one child (in households with children) were randomly selected using a Kish grid [10]. Individuals were tested for anti-HCV using the anti-HCV chemiluminescent microparticle immunoassay (CMIA) on a fully automated ARCHITECT i2000SR analyzer (Abbott Diagnostics, Wiesbaden, Germany). Positive samples were tested for HCV RNA by the Abbott RealTime HCV Assay (Abbott Molecular Inc., Des Plaines, Ilinoy (IL), United States (US)) on the Abbott m2000rt System (Abbott, Abbott Park, IL, US), and, if RNA was detected, genotyping was performed by the Abbott *Real*Time HCV Genotype II Assay with Abbott mSample Preparation System reagents (Abbott Molecular Inc., Des Plaines, IL, US) on the same Abbott m2000rt System. Final total enrolment was 7,237 adults and 1,473 children, with participation rates of 90.3% and 72.2%, respectively. Data were collected between June 2021 and October 2021 [6]. Serosurvey results were weighted by age and sex to be representative of the Georgian population.

### Mathematical model

We utilise a previously developed dynamic compartmental model of HCV transmission among PWID and the general population. The original model was parameterised and calibrated to represent the current HCV epidemic in Georgia up to 28 February 2019. The model was used to evaluate the impact of scaling up HCV treatment on the prevalence and incidence of HCV infection in Georgia over time, and to determine the levels of treatment required to reach Georgia’s HCV elimination target (90% reduction in prevalence compared with 2015 estimates) [8].

The framework of the model is based on a traditional susceptible-infected (SI) type model, with additional compartments to represent the population undergoing treatment for HCV, as described in the Supplementary Material. After treatment, the proportion of patients who are cured become susceptible to re-infection. The model is further stratified by sex (male and female), age, liver disease status, and IDU, resulting in a total of 810 differential equations. There are nine age groups (< 15, 15–17, 18–24, 25–29, 30–34, 35–39, 40–44, 45–49, and ≥ 50 years). Five liver disease stages represent none/mild liver disease, moderate liver disease, compensated cirrhosis, decompensated cirrhosis, and hepatocellular carcinoma. IDU is stratified into people who have never injected drugs, PWID, and people who used to inject drugs [8].

Individuals susceptible to HCV infection enter the model in the youngest age category and then age through the model. Some individuals start injecting drugs when they become young adults and can then cease injecting drugs to become people who used to inject drugs. Susceptible individuals become infected with HCV at a rate that is proportional to the chronic hepatitis C prevalence, with a rate of transmission applying to the whole population and an additional higher IDU-specific transmission rate applying to PWID, who also have higher HCV infection prevalence. Both transmission rates are allowed to vary over time to account for changes in risk and harm-reduction services (needle and syringe programmes (NSP), and opioid substitution therapy (OST)) intervention coverage.

Chronic HCV infection results in progression through HCV-related liver disease. Individuals with decompensated cirrhosis (DC) and hepatocellular carcinoma (HCC) experience heightened liver-related mortality [11]. Infected individuals can be treated, which if successful leads to a sustained virologic response (SVR, effective cure), resulting in individuals becoming susceptible to re-infection. SVR rate was calculated according to liver disease stage based on elimination programme data and accounting for loss to follow up (89% for mild/moderate liver disease and 86% for cirrhosis) [8]. SVR halts disease progression for individuals with mild/moderate liver disease, while it continues at a slower rate for those with compensated cirrhosis or more progressed disease. Individuals in the model can die from age-related mortality and drug-related mortality, as well as from liver disease.

The original model was parameterised and calibrated to data from the 2015 national serosurvey and bio-behavioural surveys of PWID from 1997 to 2015 and validated against data on hepatitis C incidence and prevalence in PWID as described previously [8]. In short, we simulated a stable population to approximate the current demographics, while accounting for historical changes in IDU over time. This was done by calibrating to age and sex-specific prevalence from the 2015 serosurvey [1], and age-based differences in HCV infection prevalence in PWID in 1997 compared with 2015, as well as the population size and demographic changes of PWID over the same period. These estimates were gathered from the bio-behavioural surveys and triangulated population size estimates conducted by local organisations in Georgia with extensive experience working with PWID [12-14]. The target summary statistic estimate for PWID population size was 49,700 in 2014 [14], with the model fits resulting in a higher average population size with 95% CrI overlapping the target value, 83,999 (95% CrI: 23,932 to 190,501) [8].

A total of 554 parameter sets were fit, from which median and 95% CrI were calculated for model outputs. Following model calibration, the previous analysis used monthly treatment data and SVR rates from the elimination programme for 1 May 2015 to 28 February 2019, to estimate the impact of treatment in terms of changes in incidence, prevalence, and mortality. Incidence was calculated dynamically based on the prevalence in PWID and the general population, with a force of infection that accounts for assortative mixing among PWID, harm reduction measures, and separate rates of transmission per interaction for PWID and the general population.

For this analysis, we used the same previously published model, but re-ran it with more recent treatment data available until the date of analysis. Observed treatment numbers from the HCV elimination programme database were included through 28 February 2022 (a total of 77,168 treatments initiated since 1 May 2015, averaging 941 per month). Under the programme, treatment is provided through specialised infectious disease clinics, primary healthcare clinics, and harm reduction sites to encourage treatment uptake [15,16]; as in the previous analysis [8], we assume treatments are given at the same rate to PWID and the general population.

The impact of the programme was projected forward to the end of 2030. Some model runs (n = 22; 4%) failed and were excluded; this is due to input treatment numbers exceeding the modelled number of individuals remaining in the compartment (for example, these runs tend to have much lower numbers of individuals with decompensated cirrhosis). The model was coded and run in Matlab 2021a, with output files analysed in R [17].

### Model weighting

The serosurvey results for chronic hepatitis C prevalence in 2021 were stratified into age categories (18–29, 30–39, 40–49, ≥ 50 years, all adults), as well as by sex (men, women), and reported history of IDU (ever injected). We used these eight categories to compare the model projections with the serosurvey prevalence estimates, using a modelled date of 31 July 2021 (midpoint of serosurvey).

We first evaluated whether the bounds of the 95% CrIs of the modelled prevalence projections overlapped with the 95% CIs of the 2021 serosurvey prevalence estimates for each of the eight categories. For each run, we (i) evaluated whether its projected values fell within the 95% CI of the prevalence estimate for each category from the 2021 serosurvey, and (ii) calculated the Euclidean distance between each projected value and the mean prevalence estimate.

We then used three weighting methods to assess the goodness of fit of each model run to the serosurvey data. Firstly, we filtered model runs to only include those which fall inside the 95% CI of all eight prevalence values (‘filtered model fits’). We used logistic regression to assess what differences in specific model input parameters (including birth rate, transmission rates, and timing of parameter changes) or characteristics of a model run (total, PWID and former PWID population size; overall and PWID hepatitis C incidence; overall hepatitis C prevalence, and cirrhotic population size at the time of the serosurvey) determine whether a model run is accepted by this filtering process or not. Secondly, we weighted each run by the proportion of prevalence estimates for which the model projections were within the 95% CI bounds (‘weighted by fits’). Thirdly, we scaled and normalised the calculated Euclidean distance for each measure, and assigned a weight calculated as the inverse of the mean of all the eight distances (‘weighted by distance’).

### Updated impact projections

We used the model incorporating treatment data from May 2015 to February 2022 (77,168 people treated) to estimate how the incidence and prevalence of chronic hepatitis C have decreased overall and among PWID, and to determine the cases and deaths averted. For each outcome, we compared how the new weighted projections differ from the original model projections. We projected forward treatment rates from March 2022 of 1,000 per month, 406 per month, or 5% of cases per year (which corresponds to continuing the current treatment numbers as a proportion of cases rather than number treated), to evaluate when Georgia will reach different elimination targets. The targets include: the relative targets of a 90% reduction in chronic prevalence, 80% reduction in incidence and 65% reduction in mortality, all compared with 2015 levels, and the absolute targets of decreasing hepatitis C incidence among PWID to < 2 per 100 person-years, and overall hepatitis C incidence to < 5 per 100,000 person-years [18].

### Impact of COVID-19 epidemic

The COVID-19 pandemic affected healthcare services, including enrolment in the HCV elimination programme. Screening rates in 2020 declined 25% compared with 2019 [9]. HCV treatment initiation rates from March 2020 to February 2022 were 60% lower (406/month compared with 996/month in 2019). We used this observed reduction in treatment numbers from March 2020 to estimate the reduction in cases and deaths averted compared with if treatments had continued at the average rate achieved in 2019.

## Results

### Comparison of model to serosurvey results

**Figure 1:**
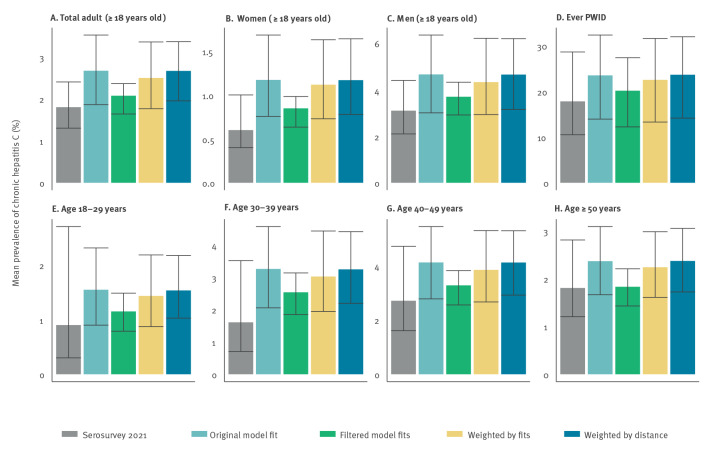
Model projections for original and weighted/filtered projections of chronic hepatitis C prevalence^a^ compared with 2021 serosurvey results^b^, by population group including (A) total adults (B) women, (C) men, (D), adults who ever injected drugs and (E–H) age categories, Georgia, 2021

Figure 1 shows that the original model projections overestimated the chronic prevalence of HCV infection in all eight groups (18–29-, 30–39-, 40–49-, ≥ 50-year-olds, all adults, men, women, and people who report a history of IDU) in 2021 as compared with the results from the 2021 serosurvey. For example, the adult chronic hepatitis C prevalence found in the 2021 serosurvey was 1.8% (95% CI: 1.3 to 2.4%) compared to the projection of 2.7% (95% CrI: 1.9 to 3.5%). However, the modelled CrIs and serosurvey CIs overlapped in all cases, with 14.3% (76/532) of all model runs lying within the CIs for all chronic hepatitis C prevalence data estimates, denoted as fitting the data. In total, 31.6% (168/532) of model runs fit the overall adult hepatitis C prevalence, 28.0% (149/532) fit the female adult prevalence, 42.5% (226/532) fit the male adult prevalence, and 85.3% (454/532) fit the hepatitis C prevalence among those having ever practiced IDU. In terms of age groups, 99.8% (531/532) fit the hepatitis C prevalence of the 18–29-year-olds, 64.8% (345/532) fit that of the 30–39-year-olds, 74.4% (396/532) that of the 40–49-year-olds, and 84.4% (449/532) that of the ≥ 50-year-olds. Of the weighting methods, the filtered model fits were closest to the 2021 serosurvey data estimates (Figure 1).

PWID: people who inject drugs.

^a^ The means are represented by the heights of the bars and whiskers represent 95% credible intervals.

^b^ The means are presented by the heights of the bars and whiskers represent 95% confidence intervals.

**Figure 2:**
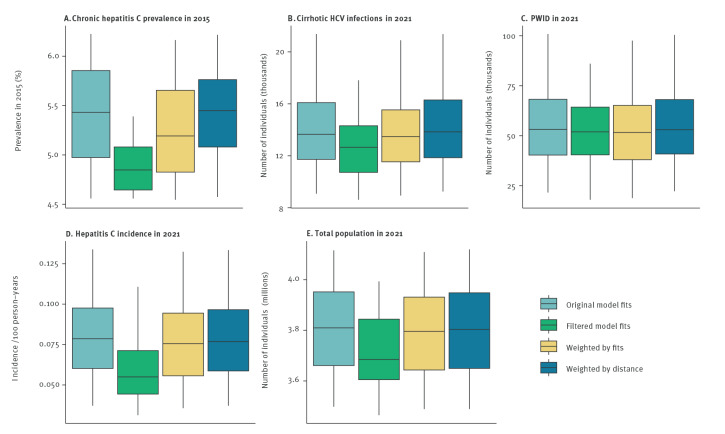
Comparison of model characteristics including (A) chronic hepatitis C prevalence, (B, C) number of cirrhotic HCV infections and PWID, (D) incidence of hepatitis C in the general population and (E) total population size, under different weighting measures, Georgia, 2015 and 2021

HCV: hepatitis C virus; PWID: people who inject drugs.

Each of the weighting methods affected key population characteristics such as prevalence of chronic hepatitis C, number of cirrhotic individuals, and total population size, with the greatest difference in the filtered model fits (Figure 2). Bivariate logistic regression comparing the model parameters for the filtered model fits with those that did not fit all data prevalence estimates found the model fits had: lower birth rate (parameter affecting population size; OR: 0.9998; 95% CI: 0.9997 to 0.9999); lower HCV transmission rate in PWID (OR: 0.01; 95% CI: 0.00 to 0.84); greater reduction in general population transmission in the late 1990s (OR: 0.03; 95% CI: 0.00 to 0.22); and later year of general population transmission reduction (OR: 2.56; 95% CI: 1.01 to 6.49). The overall result is that the filtered model runs tended to have a lower total population size and lower chronic hepatitis C prevalence in 2015 (average of 129,918 HCV infections instead of 147,639 in 2015), lower hepatitis C incidence in the general population (100 instead of 161 per 100,000 person-years in 2015), and fewer cirrhotic infections (12,965 instead of 13,483 in 2015).

Plots show median and inter-quartile range in box, and whiskers extend to 95% credible intervals.

Chronic hepatitis C prevalence in adults (≥ 18 years old) is shown for 2015, while all other measures (cirrhotic HCV infections, PWID population size, HCV infection incidence in the general population, and total population size), are shown for the date of the serosurvey in 2021.

### Updated impact projections

Using the filtered model fits, we estimated that the hepatitis C incidence on 1 March 2022 was 66 (95% CrI: 34 to 131) per 100,000 person-years overall (total population of all ages) and 1.14 (95% CrI: 0.08 to 6.4) per 100 person-years in PWID (all ages). This equates to a 57% (95% CrI: 19 to 68%) decrease in incidence since 2015 among PWID and 58% (95% CrI: 52 to 67%) decrease overall (Table). The estimated incidence for PWID in 2015 was higher in the filtered fits than for the original model fits, while all other incidence estimates were lower in the filtered model fits (Table). In terms of achieving different HCV elimination targets, the absolute incidence target has been achieved in PWID (< 2/100 person-years), but not overall population (< 5/100,000 person-years). None of the relative incidence targets have been reached by 2022.

**Table:** Reduction in hepatitis C incidence in original model and filtered fits, Georgia, 2015–2022

Model characteristic	Type of results		PWID only	Non-PWID only (general population)	Overall
Filtered to fit serosurvey	Median HCV infection incidence (95% CrI)	1 Jan 2015	2.51^a^ (0.23 to 7.90)	132^b^ (81 to 262)	161^b^ (92 to 302)
		1 Mar 2022	1.14^a^ (0.08 to 6.43)	52^b^ (30 to 107)	66^b^ (34 to 131)
	Reduction (95% CrI)		57% (19 to 68%)	60% (55 to 68%)	58% (52 to 67%)
All original model runs	Median HCV infection incidence (95% CrI)	1 Jan 2015	2.34^a^ (0.19 to 6.78)	165^b^ (82 to 273)	193^b^ (100 to 317)
		1 Mar 2022	1.17^a^ (0.07 to 5.60)	7 ^b^ (36 to 131)	92^b^ (43 to 160)
	Reduction (95% CrI)		50% (12 to 67%)	54% (44 to 64%)	53% (42 to 64%)

CrI: credible intervals; PWID: people who inject drugs.

^a^ Incidence per 100 person-years.

^b^ Incidence per 100,000 person-years.

The filtered model fits estimated that the treatments given up to March 2022 have averted 9,186 (95% CrI: 5,396 to 16,720) infections and 842 (95% CrI: 489 to 1,324) deaths, accumulating threefold for cases averted (26,154) and fivefold for deaths averted (3,971) if tracked to 2030 without additional treatments after March 2022.

**Figure 3:**
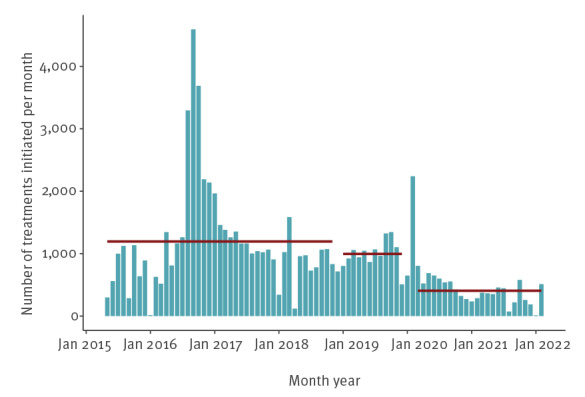
Number of individuals initiating treatment for hepatitis C per month^a^ and average numbers of treatments over certain periods^b^, Georgia May 2015–February 2022

^a^ Blue bars.

^b^ Horizontal red lines show by their position relative to the Y axis, the average number of treatments calculated over the period covered. The period covered is indicated by the respective length of the red lines in relation to the X axis. There were 1,195 treatments per month from 2015 to 2018, 996 per month in 2019, and 406 per month from March 2020 to February 2022.

**Figure 4:**
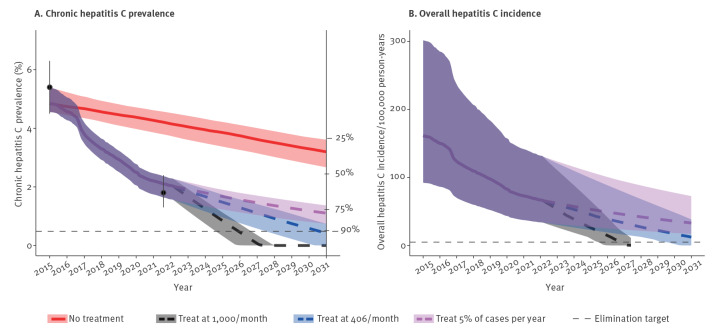
(A) Adult chronic hepatitis C prevalence and (B) overall incidence of hepatitis C over time with different treatment rates, estimated from filtered model fits, Georgia, 2015–2030

The average monthly treatment rate declined from 1,195 per month from 2015 to 2018, to 996 per month in 2019 and 406 per month over March 2020–February 2022 (Figure 3). At the current treatment rate of 406 per month, the filtered model runs projected that a 90% reduction in prevalence and incidence can be reached in 2030, while increasing treatment rates to 1,000 per month will reach this target in the beginning of 2026 (Figure 4).

HCV: hepatitis C virus.

The full red line represents the baseline with no treatment. The non-red solid lines show the numbers of treatments given through February 2022, while the dotted lines from March 2022 represent treatment at 1,000/month (black); treatment at 406/month (blue); and treatment of 5% of cases per year (purple). Black points and error bars in A show mean and 95% confidence intervals of adult chronic hepatitis C prevalence from 2015 and 2021 serosurveys.

Horizontal dashed lines show elimination targets: (panel A) 90% reduction in prevalence, (panel B) absolute incidence 5/100,000 person-years.

At the current treatment rate, the overall absolute incidence target of five per 100,000 will not be reached by 2030. However, if the treatment rate were to increase to 1,000 per month from March 2022, then this target will be reached in the last quarter of 2026. If only 5% of cases are treated per year, elimination will not be reached by 2030.

### Impact of COVID-19

If the treatment rate had not decreased to 406 per month during March 2020–February 2022 due to the COVID-19 pandemic, but instead had remained at 996 per month as in 2019, 13,344 (95% CrI: 13,236 to 13,437) more infections would have been treated, and the filtered modelled chronic hepatitis C prevalence would have been 1.6% in March 2022, instead of 2.0%. This corresponds to 438 (95% CrI: 223 to 744) more HCV infections averted (4% and 10% difference compared with estimated infections averted with achieved treatment numbers over May 2015–February 2022 and March 2020–February 2022, respectively), accumulating to 4,259 (95% CrI: 2,167 to 7,305) (15% difference in infections averted) by end of 2030, and 106 (95% CrI: −152 to 411) more deaths averted by 2030 (3% difference compared with achieved treatment numbers), without additional treatments from March 2022.

## Discussion

The national hepatitis C serosurvey conducted in 2021 provided an opportunity to reflect on the accuracy of our model of the HCV epidemic in Georgia. Although the unweighted model overestimated the chronic hepatitis C prevalence found in the 2021 serosurvey, the CrIs of the model projections all overlapped with the estimates from the serosurvey. Our analyses indicate that this could be due to our model overestimating the original number of people with chronic hepatitis C in the country in 2015. The model was fit to the full uncertainty in the serosurvey prevalence estimates in 2015, but only those runs with lower prevalence in 2015 or lower population size of Georgia were likely to fit the 2021 data. At the current treatment rate of 406 per month, a 90% reduction in hepatitis C prevalence and incidence will be reached in 2030 but the absolute elimination target of less than five per 100,000 person-years in the overall population will not be reached by 2030. Reaching these targets can be brought forward by scaling up treatment numbers. Our model suggests the absolute incidence target for PWID (< 2/100 person years) has already been reached.

Data suggest that treatment rates in Georgia decreased by ca 60% during the COVID-19 pandemic from March 2020–February 2022 compared with 2019 [9]. Our modelling estimates that this led to a 10% reduction in the impact of the elimination programme on infections averted over that period compared with if treatment rates had not reduced, and a 4% reduction in the impact of the programme over 2015–2022. COVID-19 has also had an impact on global HCV elimination efforts around the world [19]. A recent hypothetical model which assumed a pessimistic scenario of no treatment in 2020 predicted that globally this would have resulted in 121,000 excess incident cases and 623,000 excess prevalent infections over 2020–2030, with 55% of the excess infections occurring in low and middle-income countries [19].

The decrease in treatment rates that occurred during the pandemic may be due in part to difficulties in case finding as progress is made towards elimination. A decline in treatment has been observed in several countries even before the pandemic [19]. In Georgia, treatment initiation peaked in September 2016, and reaching people who have not yet been linked to care has required programmatic adjustments such as deploying patient navigators and decentralising diagnostic testing [20], as well as evaluating barriers to treatment [21]. The ‘last mile’ to elimination can be the hardest part, where an increased effort is required to identify remaining cases, and increased population susceptibility can lead to a resurgence in incident infections [22]. Similar challenges in going the last mile have been faced in Egypt, which is approaching HCV elimination after scaling up screening and treatment through a robust national programme [23].

Our model suggests that incidence is relatively low in PWID, but that the overall incidence is still well above the WHO absolute target of five per 100,000 person-years. This is because the WHO absolute target is based on a reduction of 80% from the global average incidence in 2015 [18]. In Georgia, where general population prevalence and incidence were well above the global average, reaching the absolute target would require a 97% reduction in incidence overall. The 1.8% adult prevalence found in the 2021 serosurvey is much higher than 0.7% estimated globally for 2020 [24]. For PWID, recent studies support the model’s estimated HCV incidence being lower than the global average, which a systematic review recently estimated to be 12.1 per 100 person-years [25]. For example, one pilot study conducted in 2015–2017 found incidence of reinfection after treatment to be 1.2 per 100 person-years and 8.3 per 100 person-years in a control group of individuals seeking harm reduction services [26]. The 2022 bio-behavioural survey found that 13% of all PWID who had been treated and cured (since the HCV elimination program began in 2015) had since been reinfected, but no timeframe is available for the denominator of an incidence estimate [27]. Because of the baseline 2015 low incidence, according to our model, reaching the WHO absolute incidence target only required an estimated 20% reduction in incidence for PWID. In addition, while a high proportion of the infected population are PWID, our model finds that this has been declining over time such that PWID contributed more in 2015 to the overall incidence than 2021, as illustrated in the Supplementary Material Figure 2. Additional studies to measure hepatitis C incidence would help to validate modelled estimates [28]. Our model assumptions and results about the impact of treatment on PWID are supported by evidence that PWID are being treated at high rates, with the 2022 bio-behavioural survey including self-reporting that 43% of PWID have previously taken medications for HCV, while 18.6% of the study sample and only 32% of individuals with HCV antibodies were RNA positive [27]. Comparing the 2015 and 2021 serosurveys found that the prevalence of chronic HCV infection among people who reported ever injecting drugs declined from 51.1% to 17.8% (65% decrease), a similar decline to the per cent decrease in chronic infection among the overall population (67% decrease), which was identified in the same study [6].

A key strength of this paper is the use of real-world data to update the previously validated model of HCV transmission in Georgia [8] and evaluate the path to elimination. It is unique to have a national-level model of HCV transmission which captures the scale-up of treatment achieved. Previous models of national elimination programmes rely on older data for model calibration and have not yet been updated to reflect progress in terms of HCV treatment scale-up [29-31].

This work has several limitations. Many of the input parameters to the model are uncertain, but wide uncertainty ranges have been reduced through fitting to known data points. As discussed above, it is unknown whether treatments would have been maintained at 2019 levels in absence of the COVID-19 pandemic, as treatment initiation numbers are likely to decline as a programme progresses. In addition, to fit the new prevalence estimates from the 2021 serosurvey the model parameter sets were solely re-weighted, with no changes being made to the structure of the model or assumptions updated from the original model development. This reweighting suggests that we may have overestimated the 2015 prevalence of chronic hepatitis C, although the updated estimates still fit within the 95% CI of the 2015 serosurvey. However, the impressive reduction in prevalence found in the 2021 serosurvey compared to modelled projections of the reduction in prevalence may indicate there are other differences between the model and reality that have not been accounted for. Unfortunately, we are unsure what these could be. There is a chance that the serosurvey in 2015 overestimated, or in 2021 underestimated, the true prevalence of chronic hepatitis C. However, anti-HCV prevalence did not change significantly between the surveys which indicates the estimates are reliable [6]. Population-based surveys can miss high risk groups such as PWID, however, the same methods were used in each survey and history of IDU was identified as a risk factor for HCV infection in both surveys, the proportion reporting ever injecting drugs did appear to decrease from 4.2% (95% CI: 3.4 to 5.1%) in 2015 to 3.0% (95% CI: 2.3 to 3.9%) in 2021, though the CIs overlap [6].

In addition, because of uncertainty in their benefits, we did not account for the increase in preventative measures, such as blood safety and infection control regulations, that have occurred alongside treatment during past 5 years. These may have caused further decreases in incidence but are less likely to affect prevalence.

### Conclusion

The impressive reduction in hepatitis C prevalence found between the 2015 and 2021 national serosurveys in Georgia indicate that the elimination programme, which includes a massive scale up in testing and treatment, has been highly effective at tackling the hepatitis C epidemic in the country. Our modelling projects that this reduction in prevalence translates to an impressive reduction in incidence, particularly among PWID. Ensuring continued engagement in HCV treatment is essential to achieve elimination targets.

## Supplementary Data

276000 Supplementary Material

## References

[1] Hagan LM Kasradze A Salyer SJ Gamkrelidze A Alkhazashvili M Chanturia G Hepatitis C prevalence and risk factors in Georgia, 2015: setting a baseline for elimination. BMC Public Health. 2019;19(S3) Suppl 3;480. 10.1186/s12889-019-6784-3 32326913 PMC6696670

[2] Blach S Zeuzem S Manns M Altraif I Duberg A-S Muljono DH Polaris Observatory HCV Collaborators . Global prevalence and genotype distribution of hepatitis C virus infection in 2015: a modelling study. Lancet Gastroenterol Hepatol. 2017;2(3):161-76. 10.1016/S2468-1253(16)30181-9 28404132

[3] Grebely J Larney S Peacock A Colledge S Leung J Hickman M Global, regional, and country-level estimates of hepatitis C infection among people who have recently injected drugs. Addiction. 2019;114(1):150-66. 10.1111/add.14393 30035835 PMC6657799

[4] World Health Organization. Global Health Sector Strategy on Viral Hepatitis, 2016-2021. World Health Assembly: Geneva; 2016. p. 53.

[5] Nasrullah M Sergeenko D Gvinjilia L Gamkrelidze A Tsertsvadze T Butsashvili M The Role of Screening and Treatment in National Progress Toward Hepatitis C Elimination - Georgia, 2015-2016. MMWR Morb Mortal Wkly Rep. 2017;66(29):773-6. 10.15585/mmwr.mm6629a2 28749925 PMC5657814

[6] Gamkrelidze A Shadaker S Tsereteli M Alkhazashvili M Chitadze N Tskhomelidze I Nationwide hepatitis C serosurvey and progress towards HCV elimination in the country of Georgia, 2021. J Infect Dis. 2023;jiad064. 10.1093/infdis/jiad064 36932731 PMC10506179

[7] Georgia Hepatitis Elimination Program Progress Report, 2020-2021. 2022, Ministry of Internally Displaced Persons from the Occupied Territories Labour, Health, and Social Affairs of Georgia: Tbilisi, Georgia.

[8] Walker JG Kuchuloria T Sergeenko D Fraser H Lim AG Shadaker S Interim effect evaluation of the hepatitis C elimination programme in Georgia: a modelling study. Lancet Glob Health. 2020;8(2):e244-53. 10.1016/S2214-109X(19)30483-8 31864917 PMC7025283

[9] Gamkrelidze A Handanagic S Shadaker S Turdziladze A Tsereteli M Getia V The impact of COVID-19 pandemic on the 2020 hepatitis C cascade of care in the Republic of Georgia. Public Health. 2022;205:182-6. 10.1016/j.puhe.2022.01.040 35305459 PMC9004234

[10] Kish L . A Procedure for Objective Respondent Selection within the Household. J Am Stat Assoc. 1949;44(247):380-7. 10.1080/01621459.1949.10483314

[11] Shepherd J Jones J Hartwell D Davidson P Price A Waugh N . Interferon alpha (pegylated and non-pegylated) and ribavirin for the treatment of mild chronic hepatitis C: a systematic review and economic evaluation. Health Technol Assess. 2007;11(11):1-205, iii. 10.3310/hta11110 17346498

[12] Chikovani I, Shengelia N, Sulaberidze L, Sirbiladze T, Tavzarashvili L. HIV risk and prevention behaviors among People Who Inject Drugs in seven cities of Georgia. Tbilisi, Georgia: Curatio International Foundation 2015. [Accessed 27 Nov 2019]. Available from: http://curatiofoundation.org/wp-content/uploads/2018/02/PWID-IBBS-Report-2017-ENG.pdf

[13] Kuniholm MH Aladashvili M Del Rio C Stvilia K Gabelia N Chitale RA Not all injection drug users are created equal: heterogeneity of HIV, hepatitis C virus, and hepatitis B virus infection in Georgia. Subst Use Misuse. 2008;43(10):1424-37. 10.1080/10826080802108293 18696377 PMC2825388

[14] Bemoni Public Union and Curatio International Foundation. Population Size Estimation of People who Inject Drugs in Georgia 2014. 2015; 1-51. [Accessed 7 Dec 2017]. Available from: http://curatiofoundation.org/wp-content/uploads/2016/05/PWIDs-PSE-Report-2015_ENG.pdf

[15] Dolmazashvili E Sharvadze L Abutidze A Chkhartishvili N Todua M Adamia E Treatment of hepatitis C in primary health care in the country of Georgia. Clin Liver Dis (Hoboken). 2022;20(5):175-8. 10.1002/cld.1260 36447909 PMC9700050

[16] Butsashvili M Kamkamidze G Kajaia M Gvinjilia L Kuchuloria T Khonelidze I Integration of hepatitis C treatment at harm reduction centers in Georgia-Findings from a patient satisfaction survey. Int J Drug Policy. 2020;84:102893. 10.1016/j.drugpo.2020.102893 32739613 PMC7738314

[17] R Core Team. R: A Language and Environment for Statistical Computing. 2023, R Foundation for Statistical Computing: Vienna, Austria.

[18] World Health Organization (WHO). Interim guidance for country validation of viral hepatitis elimination: Technical report. Geneva: WHO; 2021.

[19] Blach S Kondili LA Aghemo A Cai Z Dugan E Estes C Impact of COVID-19 on global HCV elimination efforts. J Hepatol. 2021;74(1):31-6. 10.1016/j.jhep.2020.07.042 32777322 PMC7411379

[20] Averhoff F Shadaker S Gamkrelidze A Kuchuloria T Gvinjilia L Getia V Progress and challenges of a pioneering hepatitis C elimination program in the country of Georgia. J Hepatol. 2020;72(4):680-7. 10.1016/j.jhep.2019.11.019 31811882 PMC7418146

[21] Chikovani I Ompad DC Uchaneishvili M Sulaberidze L Sikharulidze K Hagan H On the way to Hepatitis C elimination in the Republic of Georgia-Barriers and facilitators for people who inject drugs for engaging in the treatment program: A formative qualitative study. PLoS One. 2019;14(4):e0216123. 10.1371/journal.pone.0216123 31034530 PMC6488087

[22] Klepac P Metcalf CJ McLean AR Hampson K . Towards the endgame and beyond: complexities and challenges for the elimination of infectious diseases. Philos Trans R Soc Lond B Biol Sci. 2013;368(1623):20120137. 10.1098/rstb.2012.0137 23798686 PMC3720036

[23] Hassanin A Kamel S Waked I Fort M . Egypt’s Ambitious Strategy to Eliminate Hepatitis C Virus: A Case Study. Glob Health Sci Pract. 2021;9(1):187-200. 10.9745/GHSP-D-20-00234 33795369 PMC8087425

[24] Blach S Terrault NA Tacke F Gamkrelidze I Craxi A Tanaka J Polaris Observatory HCV Collaborators . Global change in hepatitis C virus prevalence and cascade of care between 2015 and 2020: a modelling study. Lancet Gastroenterol Hepatol. 2022;7(5):396-415. 10.1016/S2468-1253(21)00472-6 35180382

[25] Artenie A Stone J Fraser H Stewart D Arum C Lim AG HIV and HCV Incidence Review Collaborative Group . Incidence of HIV and hepatitis C virus among people who inject drugs, and associations with age and sex or gender: a global systematic review and meta-analysis. Lancet Gastroenterol Hepatol. 2023;8(6):533-52. 10.1016/S2468-1253(23)00018-3 36996853 PMC10817215

[26] Bouscaillou JKT, Le Pluart D, Butsashvili M, Labartkava K, Kamkamidze G, Inaridze I, et al. Low HCV Reinfection Rate After Treatment In People Who Infect Drugs (PWID) From A Prospective Cohort In Tbilisi, Georgia. 2018. Available from: https://www.inhsu.org/resource/low-hcv-reinfection-rate-after-treatment-in-people-who-infect-drugs-pwid-from-a-prospective-cohort-in-tbilisi-georgia/

[27] Health Research Union. Integrated Bio-Behavioral Surveillance Survey among People Who Inject Drugs. 2022: Tbilisi, Georgia.

[28] Artenie A Luhmann N Lim AG Fraser H Ward Z Stone J Methods and indicators to validate country reductions in incidence of hepatitis C virus infection to elimination levels set by WHO. Lancet Gastroenterol Hepatol. 2022;7(4):353-66. 10.1016/S2468-1253(21)00311-3 35122713 PMC10644895

[29] Lim AG Scott N Walker JG Hamid S Hellard M Vickerman P . Health and economic benefits of achieving hepatitis C virus elimination in Pakistan: A modelling study and economic analysis. PLoS Med. 2021;18(10):e1003818. 10.1371/journal.pmed.1003818 34665815 PMC8525773

[30] Lim AG Walker JG Mafirakureva N Khalid GG Qureshi H Mahmood H Effects and cost of different strategies to eliminate hepatitis C virus transmission in Pakistan: a modelling analysis. Lancet Glob Health. 2020;8(3):e440-50. 10.1016/S2214-109X(20)30003-6 32087176 PMC7295205

[31] Scott N Win TM Tidhar T Htay H Draper B Aung PTZ Hepatitis C elimination in Myanmar: Modelling the impact, cost, cost-effectiveness and economic benefits. Lancet Reg Health West Pac. 2021;10:100129. 10.1016/j.lanwpc.2021.100129 34327345 PMC8315611

