# Supplementary material for: Insights from a national survey in 2021 and from modelling on progress towards hepatitis C virus elimination in the country of Georgia since 2015
Source: Euro Surveill. 2023 Jul 27;28(30):2200952. doi: 10.2807/1560-7917.ES.2023.28.30.2200952 (PMC10375834; doi:10.2807/1560-7917.ES.2023.28.30.2200952)
Supplement: Supplementary Material [file 2200952_SupplementaryMaterial.pdf]

Supplementary Figures and Model Equations

This supplementary material is hosted by *Eurosurveillance* as supporting information alongside the article '*Insights from a national survey in 2021 and from modelling on progress towards hepatitis C virus elimination in the country of Georgia since 2015*', on behalf of the authors, who remain responsible for the accuracy and appropriateness of the content. The same standards for ethics, copyright, attributions and permissions as for the article apply. Supplements are not edited by *Eurosurveillance* and the journal is not responsible for the maintenance of any links or email addresses provided therein.

Figures

Supplementary Figure 1: Model structure schematic of previously published model used in this analysis [1]. (A) shows infection compartments; (B) shows liver disease stages, and (C) shows injecting drug use and age compartments. Gender compartments are not shown. Dashed lines show transitions to death. *Abbreviations: ex-PWID=people who used to inject drugs. Non-PWID=people who have never injected drugs. PWID=people who inject drugs.*

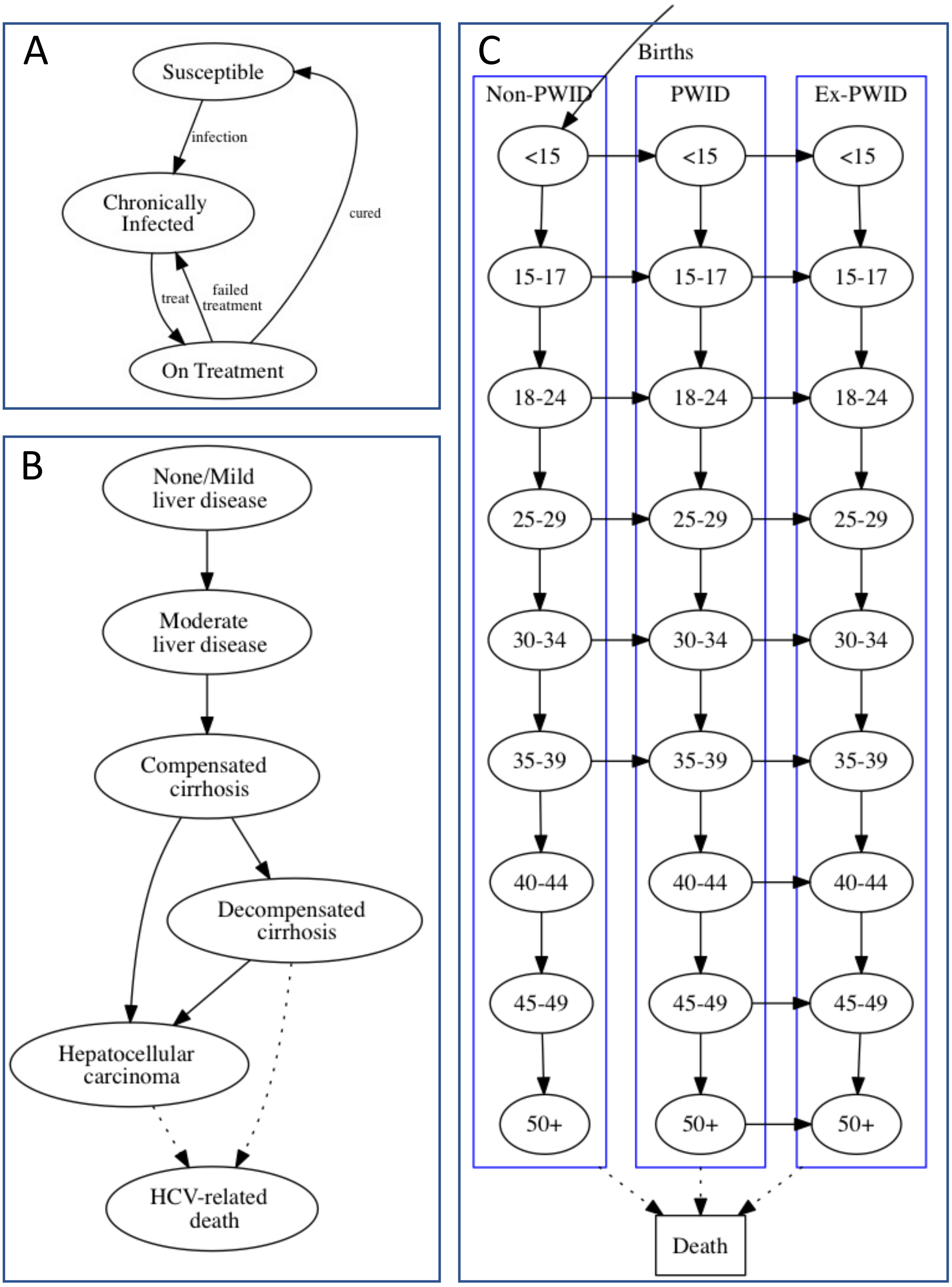

Supplementary Figure 2: Change over time in incident and prevalent hepatitis C infections in people who inject drugs (PWID) in Georgia, from modelling results: (A) Proportion of total incident hepatitis C infections in PWID over time; (B) Proportion of total prevalent hepatitis C infections in PWID (solid line) and people who used to inject drugs (dashed line)

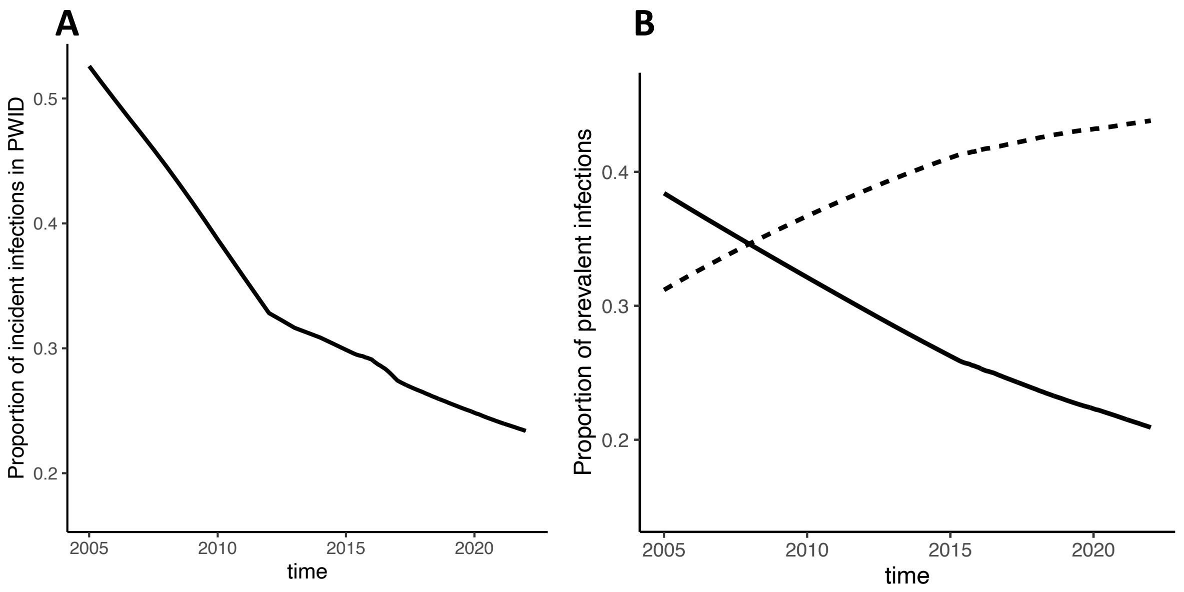

Equations

The structure of the model is written as:

$$\frac{dS_{k,n}^{i,j}(t)}{dt} = B_n^{j=1} + \left[-\left(\mu_{n,k}^{j} + \zeta^{i}\right) + \gamma_S^{i} + \chi_S^{i} + \alpha^{j} + \psi_{k=1,n}^{j}(t) + \phi_{k=2}^{j} - \lambda_k^{j}(t)\right] S_{k,n}^{i,j}(t) + \omega\pi(t) T_{k,n}^{i,j}(t)$$

$$\frac{dI_{k,n}^{i,j}(t)}{dt} = \left[-\left(\mu_{n,k}^{j} + \zeta^{i}\right) + \gamma_I^{i} + \chi_I^{i} + \alpha^{j} + \psi_{k=1,n}^{j}(t) + \phi_{k=2}^{j} - \sigma_k^{i}(t)\right] I_{k,n}^{i,j} + \lambda_k^{j}(t)\, S_{k,n}^{i,j}(t) + \omega\left(1-\pi(t)\right) T_{k,n}^{i,j}(t)$$

$$\frac{dT_{k,n}^{i,j}(t)}{dt} = \left[-\left(\mu_{n,k}^{j} + \zeta^{i}\right) + \gamma_T^{i} + \chi_T^{i} + \alpha^{j} + \psi_{k=1,n}^{j}(t) + \phi_{k=2}^{j} - \omega\right] T_{k,n}^{i,j}(t) + \sigma_k^{i}(t) I_{k,n}^{i,j}(t)$$

Where $S_{k,n}^{i,j}$, $I_{k,n}^{i,j}$, and $T_{k,n}^{i,j}$ are the number of susceptible, chronically infected, and on treatment individuals in the model, respectively. Superscript $i = 1,2,3,4,5$ represents disease progression states (1) none/mild liver disease, (2) moderate liver disease, (3) compensated cirrhosis, (4) decompensated cirrhosis, and (5) hepatocellular carcinoma. Superscript $j = 1,2 \ldots 9$ represents age groups <15, 15-17, 18-24, 25-29, 30-34, 35-39, 40-44, 45-50, and 50+, respectively. Subscript $k = 1,2,3$ represents non-PWID, PWID, and ex-PWID, respectively. Subscript $n = 1,2$ represents male and female gender, respectively. Subscript $m = S, I, T$ for parameters which vary by infection state; some parameters are also functions of time (t).

Inflows and outflows from each compartment are due to birth ($B_n^{j=1}$) and death (μ_n^j, ν_k, ζ^i), disease progression (γ_m^i, χ_m^i), aging (α^j), recruitment ($\psi_{k=1,n}^j(t)$) and cessation ($\phi_{k=2}^j$) of injecting drug use (IDU), treatment ($\sigma_k^i(t)$), and cure ($\omega, \pi(t)$). The treatment parameter $\sigma_k^i(t)$ was calculated as the ratio of number of treatments implemented per infected individuals in the model at each time point.

HCV transmission occurs through the force of infection ($\lambda_k^j(t)$). The force of infection $\lambda_k^j(t)$ is determined by the degree of assortative mixing (M) amongst PWID by age group (<30, $j \leq 4$ versus ≥30, $j \geq 5$), the impact and coverage of harm reduction measures (NSP and OST, $\varphi(t)$), general population HCV transmission parameter β, reduction in general population transmission parameter $\epsilon(t)$, and PWID HCV transmission parameter θ, where $N(t) = S(t) + I(t) + T(t)$:

For all non-PWID:

$$\lambda_{k=1,3}(t) = \beta\epsilon(t)\frac{I(t)}{N(t)}$$

For young PWID:

$$\lambda_{k=2}^{j\leq 4}(t) = \beta\epsilon(t)\frac{I(t)}{N(t)} + \theta\varphi(t)\left[(1-M)\frac{I_{k=2}(t)}{N_{k=2}(t)} + M\frac{I_{k=2}^{j\leq 4}(t)}{N_{k=2}^{j\leq 4}(t)}\right]$$

For old PWID:

$$\lambda_{k=2}^{j\geq5}(t) = \beta\epsilon(t)\frac{I(t)}{N(t)} + \theta\varphi(t)\left[(1-M)\frac{I_{k=2}(t)}{N_{k=2}(t)} + M\frac{I_{k=2}^{j\geq5}(t)}{N_{k=2}^{j\geq5}(t)}\right]$$

Where the impact of OST and NSP are determined by OST coverage ($\varrho_o(t)$), OST effectiveness (ρ_o), and NSP-associated impact $\rho_n(t)$:

$$\varphi(t) = (1 - \varrho_o(t) + \varrho_o(t)\rho_o)\rho_n(t)$$

Full details of model parameterisation can be found in the previously published supplementary material[1].

References

1. Walker, J.G., et al., *Interim effect evaluation of the hepatitis C elimination programme in Georgia: a modelling study.* Lancet Glob Health, 2020. **8**(2): p. e244-e253.
